# Supplementary figures and images for: Less intensive antileukemic therapies (monotherapy and/or combination) for older adults with acute myeloid leukemia who are not candidates for intensive antileukemic therapy: A systematic review and meta-analysis
Source: PLoS One. 2022 Feb 2;17(2):e0263240. doi: 10.1371/journal.pone.0263240 (PMC8809589; doi:10.1371/journal.pone.0263240)

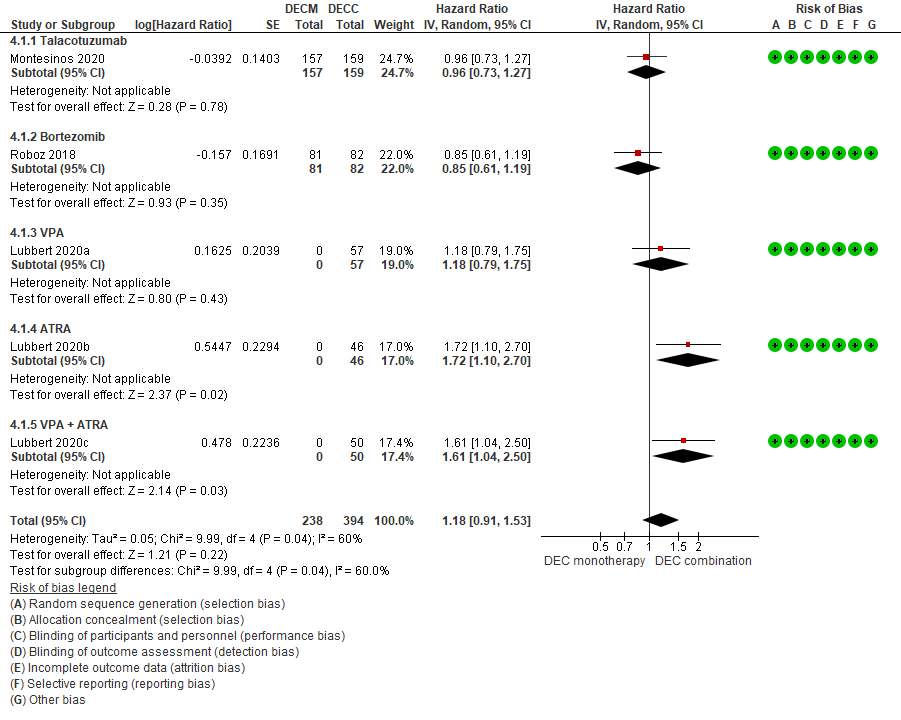

Supplement: S1 Fig — Decitabine monotherapy vs decitabine combination. (TIF) [file pone.0263240.s002.tif]

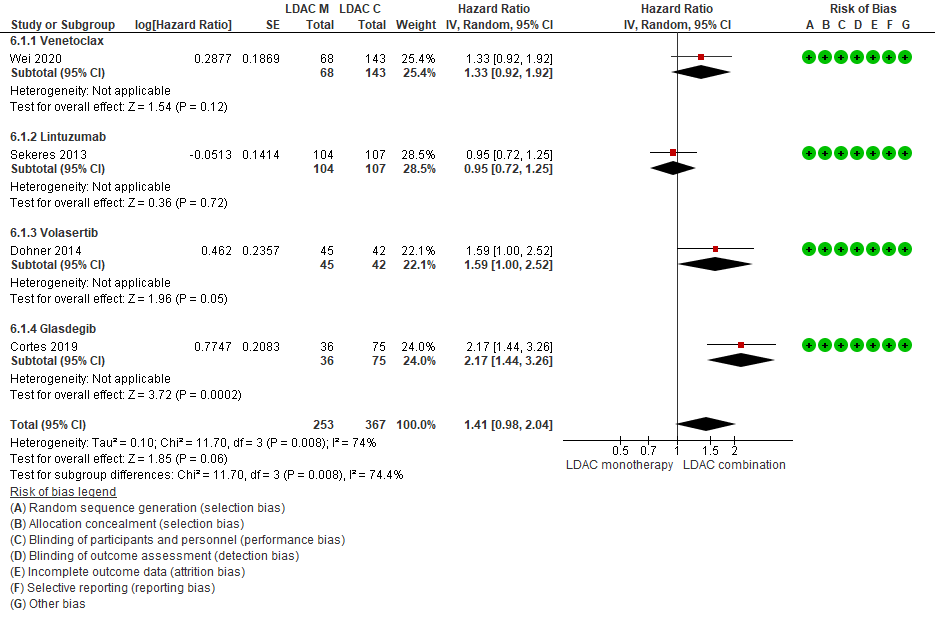

Supplement: S2 Fig — Low-dose cytarabine monotherapy vs low dose cytarabine combination. (TIF) [file pone.0263240.s003.tif]

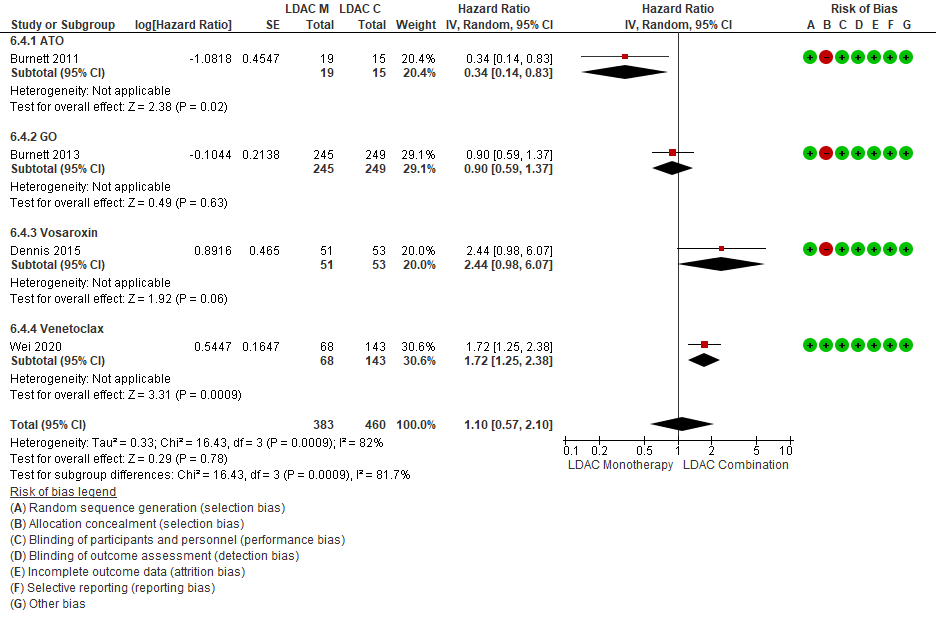

Supplement: S3 Fig — Low-dose cytarabine monotherapy vs low dose cytarabine combination. (TIF) [file pone.0263240.s004.tif]
