# Supplementary material for: Less intensive antileukemic therapies (monotherapy and/or combination) for older adults with acute myeloid leukemia who are not candidates for intensive antileukemic therapy: A systematic review and meta-analysis
Source: PLoS One. 2022 Feb 2;17(2):e0263240. doi: 10.1371/journal.pone.0263240 (PMC8809589; doi:10.1371/journal.pone.0263240)
Supplement: S1 Table — (DOCX) [file pone.0263240.s005.docx]

**S6 Table. Summary of findings table for 1-year mortality, 30-day mortality, complete remission and length of hospital stay.**

| **Table 4S - GRADE summary of findings – Other outcomes: Monotherapy or combination antileukemic therapy for older adults with AML not candidate for intensive therapy, evidence from randomized control studies and non-randomized studies.** | | | | | |
| --- | --- | --- | --- | --- | --- |
| **Comparisons** | **Relative effects and source of evidence** | **Absolute effect estimates** | | **Certainty/Quality of evidence** | **Plain languages summary** |
|  |  | **Baseline risk for control group (per 1000)** | **Difference (95% CI) (per 1000)** |  |  |
| **1 year mortality** | | | | | |
| **AZAM vs LDACM** | **RR 0.78 95% CI 0.64 – 0.94, based on 312 patients in 1 RCTs.** | **658 per 1000** | **145 fewer per 1000 (from 237 fewer to 39 fewer)** | **Low ⨁⨁◯◯**  **(Serious imprecision and indirectness)^1^** | **AZAM compared to LDACM may decrease mortality at 1 year.** |
| LDACM vs LDACC | RR 1.00 95% CI 0.92 – 1.10, based on 1060 patients in 5 RCTs. | 717 per 1000 | 0 fewer per 1000 (From 57 fewer to 72 more) | Low ⨁⨁◯◯  (Serious risk of bias and serious imprecision)^2^ | LDACM compared to LDACC may have little or no effect on 1 year mortality. |
| **LDACM vs HMA** | **HR 0.46 95% CI 0.36 – 0.59, based on 485 patients in 2 NRS,** | **518 per 1000** | **221 more per 1000 (from 160 more to 271 more)** | **Very low ⨁◯◯◯**  **( Very serious risk of bias)^3^** | **LDACM compared to HMA may increase mortality, however, we are very uncertain about this effect.** |
| **30 days mortality** | | | | | |
| DECM vs DECC | RR 1.02 95% CI 0.67 – 1.56, based on 163 patients on 1 RCT.s | 346 per 1000 | 7 more per 1000 (From 114 fewer to 194 more) | Low ⨁⨁◯◯  (Very serious imprecision)^4^ | DECM compared to DECC may have little or no effect on 30 day mortality. |
| LDACM vs LDACC | RR 0.94 95% CI 0.69 – 1.28, based on 1,171 patients in 5 RCTS. | 146 per 1000 | 9 fewer per 1000 (From 45 fewer to 41 more) | Low ⨁⨁◯◯  (Serious risk of bias and serious imprecision)^2^ | LDACM compared to LDACC may have little or no effect on 30 day mortality. |
| **Complete remission** | | | | | |
| **AZAM vs AZAC** | **HR 1.59 95% CI 1.26 – 2.00, based on 488 patients in 1 RCTs** | **717 per 1000** | **169 fewer per 1000 (From 288 fewer to 146 fewer)** | **Moderate ⨁⨁⨁◯**  **(Serious imprecision)** | **AZAM compared to AZAC probably decrease the even-free survival.** |
| LDACM vs LDACC | HR 0.91 95% CI 0.48 – 1.74, based on 843 patients in 4 RCTs. | 394 per 1000 | 30 more per 1000 (From 192 fewer to 350 more) | Very low ⨁◯◯◯  (Serious risk of bias, inconsistency and imprecision)^6^ | LDACM compared to LDACC may have little or no effect on complete remission, however, we are very uncertain about this effect. |
| AZAM vs DECM | HR 0.67 95% CI 0.40 – 1.11, based on 114 patients in 1 NRS. | 448 per 1000 | 148 fewer per 1000 (From 269 fewer to 49 more) | Very low ⨁◯◯◯  (Serious risk of bias and very serious imprecision)^7^ | AZAM compared to DECM may have little or no effect on complete remission, however, we are very uncertain about this effect. |
| **Length of Hospital stay** | | | | | |
| LDACM vs LDACC | Based on 598 patients in 2 RCTs. | Medina of 21 days. | MD 8.24 days lower (From 18.71 lower to 2.24 higher) | Low ⨁⨁◯◯  (Serious risk of bias, inconsistency and imprecision)^8^ | LDACM compared to LDACC may have little or no effect on length of hospital stay. |
| Baseline risk was obtained from the control group from the included studies.   1. We decided to rate down two levels due to imprecision and indirectness; effect estimate comes from a single study and when compared with the longest follow-up effect becomes imprecise, with no difference between the comparisons. 2. We decided to rate down two levels due to risk of bias and imprecision. More than half of the included studies had concealment bias, and the effect estimate was not consistent with benefit or harm. 3. We decided to rate down two levels due to risk of bias. Performance status is different between the treatments under comparison (ECOG: 3; 35.8% versus 0%), Intervention status is well defined but some aspects of the assignments of intervention status were determined retrospectively and is not clear if switches in treatment happen or co-interventions and is not clear if this was adjusted in the analyses and effect estimates comes from a single study. 4. We decided to rate down two levels due to imprecision. Effect estimate comes from a single study and is not consistent with benefit or harms. 5. We decided to rate down by one level due to imprecision. Effect estimate comes from a single study. 6. We decided to rate down two levels due to risk of bias, inconsistency and imprecision. More than half of the included studies had concealment bias, I2 79% (p-value 0.009) and the effect estimate was not consistent with benefit or harm. 7. We decided to rate down two levels due to risk of bias and imprecision. Researchers did not account for relevant prognostic factors in the results and effect estimate comes from a single study and the effect estimate was not consistent with benefit or harm. 8. We decided to rate down two levels due to risk of bias and imprecision. The statistical test was significant for inconsistency, however, we decided to don't downgrade because the difference was related to the effect size of the comparison. (Heterogeneity: Tau² = 48.60; Chi² = 6.00, df = 1 (P = 0.01); I² = 83%). The included studies had concealment bias and the effect estimate was not consistent with benefit or harms. | | | | | |
