# Supplementary material for: Less intensive antileukemic therapies (monotherapy and/or combination) for older adults with acute myeloid leukemia who are not candidates for intensive antileukemic therapy: A systematic review and meta-analysis
Source: PLoS One. 2022 Feb 2;17(2):e0263240. doi: 10.1371/journal.pone.0263240 (PMC8809589; doi:10.1371/journal.pone.0263240)
Supplement: S2 Table — (DOCX) [file pone.0263240.s006.docx]

**S8 Table. Overall survival subgroup analyses – summary of findings table.**

| **GRADE summary of findings –Overall survival Monotherapy or combination antileukemic therapy for older adults with AML not candidate for intensive therapy, evidence from randomized control studies.** | | | | | |
| --- | --- | --- | --- | --- | --- |
| **Comparisons** | **Relative effects and source of evidence** | **Absolute effect estimates** | | **Certainty/Quality of evidence** | **Plain languages summary** |
|  |  | **Baseline risk for control group (per 1000)** | **Difference (95% CI) (per 1000)** |  |  |
| **Decitabine monotherapy vs Decitabine combination therapy** | | | | | |
| DEC monotherapy vs Talacotuzumab + DEC. | HR 0.96, 95% CI 0.73 – 1.27, based on 316 patients on 1 RCT. | 777 per 1000 | 14 fewer per 1000 (From 111 fewer to 74 more) | Low ⨁⨁◯◯  (Very serious imprecision)^1^ | DECM compared to DECC may have little or no effect on overall survival. |
| DEC monotherapy vs Bortezomib + DEC. | HR 0.85, 95% CI 0.61– 1.19, based on 163 patients on 1 RCT. | 901 per 1000 | 41 fewer per 1000 (From 145 fewer to 35 more) | Low ⨁⨁◯◯  (Very serious imprecision)^1^ | DECM compared to DECC may have little or no effect on overall survival. |
| DEC monotherapy vs VPA + DEC. | HR 1.18, 95% CI 0.79 – 1.75, based on 57 patients in 1 RCT arm. | 825 per 1000 | 47 more per 1000 (From 77 fewer to 128 more) | Low ⨁⨁◯◯  (Very serious imprecision)^2^ | DECM compared to DECC may have little or no effect on overall survival. |
| DEC monotherapy vs ATRA + DEC | HR 1.72, 95% CI 1.10 – 2.70, based on 46 patients in 1 RCT arm. | 913 per 1000 | 72 more per 1000 (From 19 more to 86 more) | Low ⨁⨁◯◯  (Very serious imprecision)^2^ | DECM compared to DECC may decrease the overall survival. |
| DEC monotherapy vs VPA + ATRA + DEC | HR 1.61, 95% CI 1.04 – 2.50 based on 50 patients in 1 RCT arm. | 860 per 1000 | 98 more per 1000 (From 11 more to 133 more) | Low ⨁⨁◯◯  (Very serious imprecision)^2^ | DECM compared to DECC may decrease the overall survival. |
| **Low dose cytarabine monotherapy vs Low dose cytarabine combination** | | | | | |
| LDAC monotherapy vs venetoclax + LDAC | HR 1.33, 95% CI 0.92 – 1.92, based on 211 patients in 1 RCT. | 399 per 1000 | 93 more per 1000 (From 25 fewer to 225 more) | Low ⨁⨁◯◯  (Very serious imprecision)^1^ | LDACM compared to LDACC may have little or no effect on overall survival. |
| LDAC monotherapy vs Lintuzumab + LDAC | HR 0.95, 95% CI 0.72 – 1.25, based on 211 patients in 1 RCT. | 140 per 1000 | 7 fewer per 1000 (From 37 fewer to 32 more) | Low ⨁⨁◯◯  (Very serious imprecision)^1^ | LDAM compared to LDACC may have little or no effect on overall survival. |
| LDAC monotherapy vs Volasertib + LDAC | HR 1.59, 95% CI 1.00 – 2.52, based on 87 patients in 1 RCT. | 714 per 1000 | 149 more per 1000 (From 0 fewer to 243 more) | Low ⨁⨁◯◯  (Very serious imprecision)^2^ | LDACM compared to LDACC may decrease the overall survival. |
| LDAC monotherapy vs Glasdegib + LDAC | HR 2.17, 95% CI 1.44 – 3.26, based on 111 patients in 1 RCT. | 880 per 1000 | 110 more per 1000 (From 73 more to 119 more) | Low ⨁⨁◯◯  (Very serious imprecision)^2^ | LDACM compared to LDACC may decrease the overall survival. |
| DECM, decitabine monotherapy, DECC, decitabine combination. LDACM, low-dose cytarabine monotherapy, LDACC, low-dose cytarabine combination.  Baseline risk was obtained from the control group from the included studies.   1. We decided to rate down two levels due to imprecision; effect estimate comes from a single study and no consistent with benefits or harms. 2. We decided to rate down two levels due to imprecision; effect estimate comes from a single study and small sample size. | | | | | |
