# Supplementary material for: Less intensive antileukemic therapies (monotherapy and/or combination) for older adults with acute myeloid leukemia who are not candidates for intensive antileukemic therapy: A systematic review and meta-analysis
Source: PLoS One. 2022 Feb 2;17(2):e0263240. doi: 10.1371/journal.pone.0263240 (PMC8809589; doi:10.1371/journal.pone.0263240)
Supplement: S3 Table — (DOCX) [file pone.0263240.s007.docx]

**S10 Table. Relapse free survival subgroup analyses – summary of findings table.**

| **GRADE summary of findings –relapse free survival: Monotherapy or combination antileukemic therapy for older adults with AML not candidate for intensive therapy, evidence from randomized control studies.** | | | | | |
| --- | --- | --- | --- | --- | --- |
| **Comparisons** | **Relative effects and source of evidence** | **Absolute effect estimates** | | **Certainty/Quality of evidence** | **Plain languages summary** |
|  |  | **Baseline risk for control group (per 1000)** | **Difference (95% CI) (per 1000)** |  |  |
| **Low dose cytarabine monotherapy vs Low dose cytarabine combination** | | | | | |
| LDAC monotherapy vs ATO + LDAC | HR 0.34, 95% CI 0.14 – 0.83, based on 34 patients in 1 RCT. | 133 per 1000 | 86 fewer per 1000 (From 113 fewer to 21 fewer) | Low ⨁⨁◯◯  (Very serious imprecision)^1^ | LDACM compared to LDACC may increase the relapse free survival. |
| LDAC monotherapy vs GO + LDAC | HR 0.90 95% CI 0.59 -1.37, based on 494 patients in 1 RCT. | 309per 1000 | 309 fewer per 1000 (From 113 fewer to 88 more) | Low ⨁⨁◯◯  (Very serious imprecision)^1^ | LDAM compared to LDACC may have little or no effect on relapse free survival. |
| LDAC monotherapy vs Vosaroxin + LDAC | HR 2.44, 95% CI 0.98 – 6.07, based on 104 patients in 1 RCT. | 811 per 1000 | 172 more per 1000 (From 6 fewer to 189 more) | Low ⨁⨁◯◯  (Very serious imprecision)^2^ | LDACM compared to LDACC may decrease the relapse-free survival. |
| LDAC monotherapy vs Venetoclax + LDAC | HR 1.72, 95% CI 1.25 – 2.38, based on 211 patients in 1 RCT. | 189 per 1000 | 113 more per 1000 (From 41 more to 203 more) | Low ⨁⨁◯◯  (Very serious imprecision)^2^ | LDACM compared to LDACC may decrease the overall survival. |
| LDACM, low-dose cytarabine monotherapy, LDACC, low-dose cytarabine combination. ATO, Arsenic trioxide, GO, Gemtuzumab ozogamicin.  Baseline risk was obtained from the control group from the included studies.   1. We decided to rate down two levels due to imprecision; effect estimate comes from a single study and no consistent with benefits or harms. 2. We decided to rate down two levels due to imprecision; effect estimate comes from a single study and small sample size. | | | | | |
