# Supplementary material for: Less intensive antileukemic therapies (monotherapy and/or combination) for older adults with acute myeloid leukemia who are not candidates for intensive antileukemic therapy: A systematic review and meta-analysis
Source: PLoS One. 2022 Feb 2;17(2):e0263240. doi: 10.1371/journal.pone.0263240 (PMC8809589; doi:10.1371/journal.pone.0263240)
Supplement: S1 Appendix — (DOCX) [file pone.0263240.s008.docx]

**S2 Appendix. Eligibility criteria and study characteristics.**

**Type of studies**

We included randomized clinical trials (RCTs) and comparative non-randomized studies (NRS) *-prospective and retrospective observational studies, before and after studies, and studies where the comparator was a historical cohort-* comparing the outcomes of any two of the eligible treatment strategies in older patients with newly-diagnosed AML, published in any language.

**Type of participants**

We included adults 55 years old or older. We included patients with de novo AML, treatment related AML, secondary AML, acute myeloid leukemia, acute myelogenous leukemia, acute granulocytic leukemia, acute myeloblastic leukemia, acute myelocytic leukemia, acute nonlymphoblastic leukemia, acute nonlymphocytic leukemia, acute monomyelocytic leukemia, acute erythroleukemia, acute megakaryocytic leukemia. We excluded studies if patients had acute promyelocytic leukemia, or myeloid proliferations related to Down syndrome. We included studies if they enrolled participants of any age, but report separate results for those 55 years or older. We included studies if they enrolled participants of any age and present the results combined for all, but 75% or more of the participants are 55 years or older

**Type of interventions**

We included studies in which researchers compared any of the following 5 treatment strategies to another:

- Gemtuzumab ozogamicin monotherapy or in combination with any other agent.
- Low-dose cytarabine (20 mg twice daily for 10 to 14 days every 4 weeks, or 75 mg/m2 for 7 days every 4 weeks) monotherapy or in combination with other agents.
- Azacitidine monotherapy or in combination with other agents
- 5-day or 10-day Decitabine monotherapy or in combination with other agent.

We included studies in which any of these treatments were administered as the main antileukemic therapy drug, regardless of the secondary agent in the case of combinations.

We excluded studies in which researchers combined any of the interventions of interest with any agent considered part of intensive antileukemic therapy regimens.

We will exclude studies in which researchers combined any of the interventions of interest with any of the following. These can also be labeled as “intensive” antileukemic therapy.

- “7+3 therapy” (daunorubicin 90 mg/m2 or daunorubicin 60 mg/m2 + cytarabine 100mg/m2, 200 mg/m2, 400 mg/m2) with or without the addition of a third agent (gemtuzumab ozogamicin or midostaurin) or a myeloid growth factors (granulocyte colony-stimulating factor [G-CSF], granulocyte-monocyte colony-stimulating factor [GM-CSF])
- Clofarabine
- Other agents such as: FLAG (fludarabone + cytarabine), CLAG (cladribine + cytarabine), vorinostat + idarubicin + cytarabine, Bortezomib + daunorubicin + cytarabine, Lenalidomide, Modulators of drug efflux (cyclosporine, valspodar)

**Type of outcomes**

We included studies in which the researchers measured mortality, quality of life, functional status, recurrence, morphologic complete remission, severe toxicity (CTC adverse effects grade 3 or higher), or burden on caregivers, at any time point.
