# Supplementary material for: Less intensive antileukemic therapies (monotherapy and/or combination) for older adults with acute myeloid leukemia who are not candidates for intensive antileukemic therapy: A systematic review and meta-analysis
Source: PLoS One. 2022 Feb 2;17(2):e0263240. doi: 10.1371/journal.pone.0263240 (PMC8809589; doi:10.1371/journal.pone.0263240)
Supplement: S2 Appendix — (DOCX) [file pone.0263240.s009.docx]

**S3 Appendix – Search strategy items**

**OVID Medline Epub Ahead of Print, In-Process & Other Non-Indexed Citations, Ovid MEDLINE(R) Daily and Ovid MEDLINE(R) 1946 to August 2021 – (8,576 records)**

1. exp leukemia, myeloid, acute/

2. acute myeloid leukemia.mp.

3. acute myelogenous leukemia.mp.

4. acute nonlymphocytic leukemia.mp.

5. exp Leukemia, Myelomonocytic, Acute/

6. or/1-5

7. (acut$ or akut$ or agud$ or aigu$).tw,kf,ot.

8. ((promyelocyt$ or promielocitic$ or promyelozyt$ or progranulocyt$) and (leuk?em$ or leuc$)).tw,kf,ot.

9. 7 and 8

10. LEUKEMIA, MYELOID/

11. ACUTE DISEASE/

12. 10 and 11

13. (acut$ or akut$ or agud$ or aigu$).tw,kf,ot.

14. ((myelo$ or mielo$ or nonlympho$ or granulocytic$) and (leuk?em$ or leuc$)).tw,kf,ot.

15. 13 and 14

16. 9 or 12 or 15

17. 6 or 16

18. exp aged/

19. health services for the aged/ or homes for the aged/ or long-term care/ or nursing care/ or exp nursing homes/

20. (advanced years or ageing or aging or elder? or elderly or frail or geriatric? or gerontolog$ or later life or nursing care or nursing home? or old age or oldest old or pensioner? or post-menopausal or postmenopausal or senior or seniors).tw.

21. (aged or aging or ageing or elder$ or geriatric$ or gerontolog$).jw,nw.

22. ('65 year$' or 'over 65' or 'over 70' or 'over 75' or 'over 80' or 'over 85' or '85 year$').tw.

23. or/18-22

24. randomized controlled trial.pt.

25. randomized.mp.

26. placebo.mp.

27. or/24-26

28. Case-Control Studies/ or Control Groups/ or Matched-Pair Analysis/ or ((case* adj5 control*) or (case adj3 comparison*) or control group*).ti,ab.

29. cohort studies/ or longitudinal studies/ or follow-up studies/ or prospective studies/ or retrospective studies/ or cohort.ti,ab. or longitudinal.ti,ab. or prospective.ti,ab. or retrospective.ti,ab.

30. Non-randomi$ed.ab.

31. nonrandomi$ed.ab.

32. (Cohort adj study).ab.

33. (Observational adj study).ab.

34. (Case-control adj study).ab.

35. exp Survival Analysis/

36. or/28-35

37. 27 or 36

38. antileukemic therapy.mp.

39. chemotherapy.mp.

40. maintenance therapy.mp.

41. post?remission therapy.mp.

42. transfusion.mp.

43. induction.mp.

44. consolidation.mp.

45. or/38-44

46. 17 and 23 and 37

**Embase (1974 to August 2021) – (7,039 records)**

1. exp acute myeloid leukemia/

2. acute myeloid leukemia.mp.

3. acute myelogenous leukemia.mp.

4. acute nonlymphocytic leukemia.mp.

5. or/1-4

6. (acut$ or akut$ or agud$ or aigu$).tw,kw,hw,ot.

7. ((promyelocyt$ or promielocitic$ or promyelozyt$ or progranulocyt$) and (leuk?em$ or leuc$)).tw,kw,hw,ot.

8. 6 and 7

9. myeloid leukemia/

10. acute disease/

11. 9 and 10

12. (acut$ or akut$ or agud$ or aigu$).tw,kw,hw,ot.

13. ((myelo$ or mielo$ or nonlympho$ or granulocytic$) and (leuk?em$ or leuc$)).tw,kw,hw,ot.

14. 12 and 13

15. 8 or 11 or 14

16. 5 or 15

17. exp aged/

18. exp elderly care/

19. (advanced years or ageing or aging or elder? or elderly or frail or geriatric? or gerontolog$ or later life or nursing care or nursing home? or old age or oldest old or pensioner? or post-menopausal or postmenopausal or senior or seniors).tw.

20. (old$ adj3 (adult? or female? or male? or men or people or person or women)).tw.

21. ('65 year$' or 'over 55' or 'over 65' or 'over 70' or 'over 75' or 'over 80' or 'over 85' or '85 year$').tw.

22. (aged or aging or ageing or elder$ or geriatric$ or gerontolog$).jw.

23. or/17-22

24. random:.tw.

25. placebo:.mp.

26. double-blind:.tw.

27. or/24-26

28. exp cohort analysis/

29. exp longitudinal study/

30. exp prospective study/

31. exp follow up/

32. cohort$.tw.

33. exp case control study/

34. (case$ and control$).tw.

35. or/28-34

36. 27 or 35

37. 16 and 23 and 36
