# Supplementary material for: Less intensive antileukemic therapies (monotherapy and/or combination) for older adults with acute myeloid leukemia who are not candidates for intensive antileukemic therapy: A systematic review and meta-analysis
Source: PLoS One. 2022 Feb 2;17(2):e0263240. doi: 10.1371/journal.pone.0263240 (PMC8809589; doi:10.1371/journal.pone.0263240)
Supplement: S3 Appendix — (DOCX) [file pone.0263240.s010.docx]

**S4 Appendix. Study characteristics**

| **Table 1S - Study characteristics** | | | | |
| --- | --- | --- | --- | --- |
| Author, year  Sample size | Overall cytogenetics  (intermediate or poor)  N (%) | Overall Performance status  N (%) | Overall white cell count  Median (IQR) | Trial location, source of funding and clinical trial registry |
| Randomized controlled trial | | | | |
| Wei,  2020  N=211 | Intermediate: 133/211 (63)  Poor: 67/211 (31.7) | ECOG  0: 33/211 (15.6)  1: 75/211 (35.5)  2: 88/211 (41.7)  3: 15/211 (7.1) | Not reported | United states, Japan, China, Greece, Russia, Spain, France, Canada, Hungary, Korea, Czech Republic, Germany, Australia, New Zealand, Taiwan, United Kingdom, Brazil, South Africa, Belgium, Norway, Puerto Rico, Ireland, Argentina and Mexico.  Supported by AbbVie and Genentech.  NCT03069352 |
| Di Nardo,  2020  N=433 | Intermediate: 271/431 (62.8)  Poor: 160/431 (37.2) | ECOG  0 - 1: 238/431 (55.22)  2 - 3: 193/431 (44.78) | Not reported | Australia, Austria, Belgium, Brazil, Canada, China, Croatia, Czech Republic, Denmark, Finland, France, Germany, Hungary, Israel, Italy, Japan, Norway, Poland, Portugal, Russia, South Africa, South Korea, Sweeden, Taiwan, Turkey and United States of America.  Supported by AbbVie and Genentech  NCT02993523 |
| Lubbert,  2020  N= 200 | Intermediate-I; 57 (28.5)  Intermediate-II: 50 (25)  Adverse: 60 (30) | ECOG  0 = 38 (19.5)  1 = 122 (61)  2 - 3 = 40 (20) | 4.1  (1.7 – 17.5) | Germany  German Federal Ministry of Education and Research  NCT00867672 |
| Montesinos, 2020  N = 316 | Intermediate-I: 112 (35.4)  Intermediate-II: 65 (20.6)  Adverse: 104 (32.9) | ECOG  0 = 59 (18.7)  1 = 134 (42.4)  2 = 123 (38.9) | Not reported | Australia, Belgium, France, Germany, Israel, Korea, Poland, Russian federation, Spain, Sweden, Taiwan, Turkey, United Kingdom, Unites States of America.  Janssen Research & Development  NCT02472145 |
| Cortes,  2019  N = 132 | Intermediate I: 38 (32.7)  Intermediate II: 29 (25)  Adverse: 41/116 (35.3) | ECOG  0 = 14 (10.6)  1 = 47 (35.6)  2 = 70 (53.0) | Not reported | Canada, Germany, Italy, Poland, Spain and United States of America  Pfizer  NCT01546038 |
| Roboz,  2018  N= 165 | Intermediate-II: 41 (27.9)  Adverse: 58 (39.5) | EGOC  0 = 43 (26.4)  1 = 89 (54.6)  2 = 27 (16.6)  3 = 4 (2.5) | 13.3^1^  (0.4 – 212.7) | United States of America  National Cancer Institute, Alliance for Clinical Trials in Oncology. Millennium Pharmaceuticals  NCT02203773 |
| Craddock,  2017  N= 260 | Intermediate risk: 109 (42)  Poor risk: 54 (21) | ECOG  0 = 84 (32)  1 = 133 (51)  2 = 26 (10) | 14.1^1^  (24.6)^2^ | United Kingdom  Bloodwise Trials Acceleration Program  NCT not reported  ISRCTN68224706 |
| Montalban bravo,  2017  N=79 | Patients were selected based on intermediate-2 or higher risk by International Prognostic  Scoring System | ECOG  0 – 1 = 66 (83.6)  2 = 6 (7.6)  >3 = 7 (8.8) | Not reported | United States of America  Merck Sharp and Dohme Corporation supported this clinical trial  NCT00948064 |
| Dennis,  2015  N = 104 | Intermediate: 53 (50.9)  Adverse: 17 (16.3) | ECOG  0 = 18 (17.3)  1 = 67 (64.4)  2 = 18 (17.3)  3 = 1 (1) | Not reported | Denmark and United Kingdom  Sunesis Pharmaceuticals Inc.  NCT01191801  SRCTN40571019 |
| Dohner,  2014  N = 87 | Intermediate I/II: 46 (58.9)  Adverse: 28 (32) | 0 = 26 (29.8)  1 = 43 (49.4)  2 = 18 (20.6) | Not reported | Austria, Belgium, Canada, France, Germany, Italy and Norway.  Boehringer Ingelheim Pharma GmbH & Co. KG.  NCT00804856 |
| Dombret,  2014  N= 399 | Patients were selected based on intermediate-or  poor-risk cytogenetics (NCCN 2009 criteria) | ECOG  0 – 1 = 309 (77.4)  2 = 90 (22.5) | Not reported | Australia, Austria, Belgium, Canada, China, Czechia, France, Germany, Israel, Italy, Korea, Netherlands, Poland, Russian federation, Spain, Taiwan, United Kingdom and United States of America.  Editorial assistance was provided by Brian Kaiser and Sheila Truten of Medical Communication Company, Inc., funded by Celgene Corporation.  NCT01074047 |
| Prebet,  2014  N = 149 | Intermediate: 22 (14.7)  High risk: 55 (36.9) | Not measured | Not reported | United States of America  Supported by R01 CA125563501 (S.G.), R6034-08 from the Leukemia and Lymphoma Society of America  NCT00313586. |
| Burnett,  2013  N = 495 | Intermediate: 223 (45)  Adverse: 102 (20.6) | WHO  Grade 0 = 187 (37.7)  Grade 1 = 251 (50.7)  Grade 2 = 42 (8.4)  Grade 3 = 13 (2.6)  Grade 4 = 2 (0.4) | Not reported | United Kingdom  Cancer Research UK for research funding, Wyeth for the provision of gemtuzumab ozogamicin, trial staff at the Birmingham Clinical Trials Unit performed the trial management activity.  NCT not reported |
| Sekeres,  2013  N= 211 | Standard risk: 88 (41.7)  High risk: 87 (42.1) | ECOG  0 = 15 (7)  1 = 101 (48)  2 = 95 (45) | 4.3  (0 – 74) | United States of America  Seattle Genetics, Inc.  NCT00528333 |
| Kantarjian,  2012  N= 457 | Intermediate risk: 306 (63.4)  Poor risk: 174 (36) | ECOG  0 – 1 = 348 (76.1)  2 = 109 (23.8) | Not reported | United States of America France, Poland, Czech Republic, Taiwan, Canada, Australia  Hagop M. Kantarjian, Celgene, Eisai; Jiri Mayer, Eisai; Rena Buckstein, Celgene, Novartis  NCT not reported |
| Burnett,  2011  N= 166 | Intermediate: 89 (53.6)  Adverse: 32 (19.3) | WHO  Grade 0 = 49 (29.5)  Grade 1 = 95 (57.2)  Grade 2 = 13 (7.8)  Grade 3 = 8 (4.8)  Grade 4 = 1 (0.6) | Not reported | United Kingdom  Cancer Research UK (fund) and Cephalon (provided drug)  NCT not reported |
| Fenaux,  2010  N= 34 | Intermediate: 27 (79.4)  Unfavorable: 6 (14.6) | ECOG  0 = 17 (50)  1 = 16 (47)  2 = 0 | Not reported | France, United Kingdom, Sweden, Italy, Spain, Australia, United States of America  Celgene, Summit, NJ.  NCT not reported |
| Non-Randomized trials | | | | |
| Talati,  2020  N = 346 | Adverse: 121/346 (34.9)  Intermediate: 194/346 (56) | 0 - 1: 290/343 (84.5)  2 - 4: 53/343 (15.4) | 3.3  (0.2- 230.7) | United States of America  National Institutes of Cancer grant 5R01CA168677-02 (PI – Martine Extermann, MD, PhD) and in part by the Biostatistics Core Facility at the Moffitt Cancer Center & Research Institute, a National Cancer Institute-designated Comprehensive Cancer Center (P30CA076292-16) |
| Kanakasetty,  2019  N = 139 | Intermediate: 51 (36.6)  Unfavorable : 50 (35.9) | 0 = 32 (22)  1 = 90 (62)  2 = 23 (16) | Not reported | India  Not reported |
| Di Nardo  2019  N = 145 | Intermediate risk; 74 (51)  Poor risk; 71 (49) | 0 = 32 (22)  1 = 90 (62)  2 = 23 (16) | Not reported | United State of America  Pharmaceutical, and A.L. was supported by P01 grant 5P01CA066996-20 from the National Institutes of Health, National Cancer Institute. |
| Di Nardo,  2018  N = 45 | 45 (100) | 0 = 6 (13.3)  1 = 31 (69)  2 = 8 (17.7) | Not reported | United State of America  AbbVie and Genentech |
| Boddu,  2017  N = 406 | Intermediate: 61 (15)  Adverse: 241 (59.3) | 0 – 1 = 297 (73.1)  2 = 52 (12.8) | Not reported | United State of America  University of Texas and National Cancer Institute |
| Nanah,  2017  N = 56 | Intermediate: 35 (62)  Poor: 20 (36) | Not measured | Not reported | United State of America  Not reported |
| Jacob,  2015  N= 30 | Unsatisfactory: 15 (50) | 1 = 13 (43.3)  2 = 17 (56.7) | Not reported | India  The authors declare that there is no conflict of interests regarding the publication of this paper. |
| Smith.  2014  N= 487 | Not reported | Not measured | Not reported | United State of America  Celgene. |
| Quintas-Cardama,  2012  N = 114 | Focused on specific genetic defects. | 0 – 2 = 108 (94.7) | Not reported | United State of America.  The publication costs of this article were defrayed in part by page charge payment. Therefore, and solely to indicate this fact, this article is hereby marked ‘‘advertisement’’ in accordance with 18 USC section 1734. |
| Di Febo,  2007  N = 28 | Intermediate: 11 (39.3)  Adverse: 1 (3.5) | Not measured | Not reported | India  Not reported |
| 1 = Mean, 2 = Standard deviation. | | | | |
