# Supplementary material for: Less intensive antileukemic therapies (monotherapy and/or combination) for older adults with acute myeloid leukemia who are not candidates for intensive antileukemic therapy: A systematic review and meta-analysis
Source: PLoS One. 2022 Feb 2;17(2):e0263240. doi: 10.1371/journal.pone.0263240 (PMC8809589; doi:10.1371/journal.pone.0263240)
Supplement: S4 Appendix — (DOCX) [file pone.0263240.s011.docx]

**S5 Appendix – Risk of bias of the included studies**

**Table 3S. Risk of bias of included RCTs**

| **Table S3 - Risk of bias of RCTs - Modified Cochrane tool** | | | | | | | |
| --- | --- | --- | --- | --- | --- | --- | --- |
| **Author,**  **year** | **Sequence Generation** | **Allocation Sequence Concealment** | **Blinding**  **(Performance bias)** | **Blinding**  **(Outcome measurement)** | **Missing Outcome Data** | **Selective reporting** | **Comments** |
| Wei,  2020 | Low | Low | Low | Low | Low | Low | Interactive response technology was used to randomization and treatment delivery. |
| Di Nardo,  2020 | Low | Low | Low | Low | Low | Low | Interactive response technology was used to randomization and treatment delivery. |
| Lubbert,  2020 | Low | Low | Low | Low | Low | Low | Center-stratified block randomization with randomly varying block size and a ratio of 1:1:1:1 to the four treatment arms was performed centrally (Clinical Trials Unit Freiburg), in order to guarantee concealment of treatment allocation. |
| Montesinos,  2020 | Low | Probably low | Probably low | Low | Low | Low | None |
| Cortes,  2019 | Low | Probably low | Low | Low | Low | Low | Randomization technique not reported, however, interactive voice system was used, its very likely that the randomization was done by a software |
| Roboz,  2018 | Low | Probably low | Low | Low | Probably low | Low | None |
| Montalban bravo,  2017 | Probably low | Probably high | Low | Low | Probably low | Low | No allocation concealment described, adaptive randomization based on results, so likely to be predicted |
| Craddock,  2017 | Low | Probably low | Low | Low | Probably low | Low | None |
| Dennis,  2015 | Probably low | High | Low | Low | Probably low | Low | With the pick a winner design is likely that some knowledge of treatments was known |
| Dohner,  2014 | Probably low | Probably low | Low | Low | Low | Low | None |
| Dombret,  2014 | Low | Low | Low | Low | Low | Low | None |
| Prebet,  2014 | Probably low | Low | Low | Low | Low | Low | None |
| Sekeres,  2013 | Low | Probably low | Low | Low | Low | Low | None |
| Burnett,  2013 | Probably low | High | Low | Low | Probably low | Probably low | With the pick a winner design is likely that some knowledge of treatments was known |
| Kantarjian,  2012 | Low | Probably low | Low | Low | Low | Probably low | None |
| Burnett,  2011 | Probably low | High | Low | Low | Probably low | Probably low | No method for allocation concealment described nor it can be inferred that it was done properly |
| Fenaux,  2010 | Low | Low | Low | Low | Low | Low | None |

**Table 4S. Risk of bias of included NRS**

| **Table 4S - Cochrane Risk of Bias Assessment Tool for Non-Randomized Studies of Interventions**  **(ROBINS-1)** | | | | | | | | |
| --- | --- | --- | --- | --- | --- | --- | --- | --- |
| Author, year. | Bias due to confounding | Bias in selection of participants into the study | Bias in classification of interventions | Bias due to deviations from intended interventions | Bias due to missing data | Bias in measurement of outcomes | Bias in selection of the reported result | Comments |
| Talati, 2020 | Serious | Low | Moderate | Moderate | Low | Low | Low | Clinical covariates were not appropriate controlled for, with clinically meaningful difference between the comparisons (HMA previous therapies and type of AML). Intervention status is well defined but some aspects of the assignments of intervention status were determined retrospectively. The center was part of different clinical trials and other changes in practice over time, so bias due to deviation from the intended intervention is expected, as switches and co-interventions. |
| Kanakasetty, 2019 | Serious | Low | Moderate | Serious | Low | Low | Low | Performance status is different between the treatments under comparison (ECOG: 3; 35.8% versus 0%).  Intervention status is well defined but some aspects of the assignments of intervention status were determined retrospectively.  is not clear if Switches in treatment happen or co-interventions and is not clear if this was adjusted in the analyses. |
| Di Nardo 2019 | Serious | Low | Low | Moderate | Low | Low | Low | Some of the covariates were not equally distribute among the participants (e. g. Hydroxyurea before study initiation).  The interventions related to the second agent might influence the treatment in the comparisons; Different proportions of patients in each group received granulocyte colony-stimulating factor or prophylactic non-azole antifungal agents. Venetoclax dose could be modified according to toxicity. |
| Di Nardo, 2018 | Serious | Low | Low | Moderate | Low | Low | Low |  |
| Boddu, 2017 | Serious | Low | Moderate | Moderate | Low | Low | Low | Patients differed regarding some potential prognostic factors: creatinine, WBC count, PS.  HMA based includes Monotherapy and Combination, unclear which one is which.  HMA groups could not have received secondary agents that increased their efficacy |
| Nanah, 2017 | Serious | Low | Low | Moderate | Low | Low | Low | There is no comparison of baseline characteristics that could influence the outcomes between the groups, nor the results are adjusted for them.  Patients in both groups could receive secondary agents, so differences could arise |
| Jacob, 2015 | Serious | Serious | Low | Low | Low | Low | Low | The authors reported some confounders in table one, however, they don’t describe the population in detail, like comorbid.  Selection into the study was related to intervention and outcome (Case-control study) |
| Smith. 2014 | Serious | Low | Low | Low | Low | Low | Low | Researchers did not account for relevant prognostic factors in the results. |
| Quintas-Cardama, 2012 | Serious | Low | Low | Moderate | Low | Low | Low | Researchers did not account for relevant prognostic factors in the results (ECOG performance status and Median WBC were different between the groups, affection the prognosis at baseline between the comparisons)  No information provided, No description of the co-interventions and possible effect modifiers in the treatments of interest |
| Di Febo, 2007 | Serious | Moderate | Low | Low | Low | Low | Low | In the ARA-C+ATRA group half of the population was post MDS related AML and presented previous malignancy, the study does not assess time-varying confounding. No confounding stratification in the analysis.  Unclear if all participants eligible in both groups were included- number may be too Low for the amount of time that the study was run |
