# Supplementary material for: Less intensive antileukemic therapies (monotherapy and/or combination) for older adults with acute myeloid leukemia who are not candidates for intensive antileukemic therapy: A systematic review and meta-analysis
Source: PLoS One. 2022 Feb 2;17(2):e0263240. doi: 10.1371/journal.pone.0263240 (PMC8809589; doi:10.1371/journal.pone.0263240)
Supplement: Attachment — Submitted filename: Response to Reviewers.docx [file pone.0263240.s013.docx]

14^th^ January 2022

Dr. Mona Pathak, PhD

Academic Editor

Plos One

Dear Dr. Pathak,

Thank you for taking the time to review our manuscript ID PONE-D-21-13321, and for all the thoughtful feedback. Please see below our responses (in red) to the comments regarding our manuscript, “Less intensive antileukemic therapies (monotherapy and/or combination) for older adults with acute myeloid leukemia who are not candidates for intensive antileukemic therapy: a systematic review and meta-analysis”. All changes to the manuscript are tracked.

Please let us know if anything requires further clarification.

Sincerely,

Luis Enrique Colunga Lozano

On behalf of the authors

Journal Requirements:

Please review your reference list to ensure that it is complete and correct. If you have cited papers that have been retracted, please include the rationale for doing so in the manuscript text, or remove these references and replace them with relevant current references. Any changes to the reference list should be mentioned in the rebuttal letter that accompanies your revised manuscript. If you need to cite a retracted article, indicate the article’s retracted status in the References list and also include a citation and full reference for the retraction notice.

We reviewed our reference list. We did not identify any retracted papers and the citations are correct.

Additional Editor Comments:

Thank you for revision and addressing the raised concerns, the manuscript is more clear in contents after the revisions. Still, it will be great if authors could provide an insight on the reported differential effectiveness of less intensive anti-leukemic therapies.

Thanks for your suggestion. We added the following paragraph in our discussion in order to provide insights into the comparisons.

Therapy selection for older adults with AML who are not candidates for intensive antileukemic therapy is based on the patient fitness, patients’ characteristics (cytogenic and molecular profiles), the trade-off between drug safety and toxicity, and patients' values and preferences. The scientific community agrees on offering therapies based on HMA agents (e.g., azacytidine, decitabine) with some exceptions: liver and kidney severe disease, prior HMA therapy, and the presence of an actionable mutation. For these populations other options are available (e.g., Low-dose cytarabine). Currently, combination therapy has become the standard of care for these patients. However, the secondary agent depends on their availability in each setting and the presence of specific genetic mutations. Venetoclax (BCL2 inhibitor) is the preferred secondary agent to add to the HMA therapies, this is based on promising results from NRS and RCTs (mentioned previously). In our review, we identify benefits from the combination therapy with venetoclax. However, the certainty of the effect was judged to be low after creating a pooled estimate (imprecision and inconsistency). The same situation was identified with other secondary agents. We are aware that creating pooled estimates without stratifying based on the second agent may impact the effect estimate of a specific agent (e.g., venetoclax). In the comparison with enough studies, we undertook a subgroup analysis to explore their effect. However, the AZA monotherapy vs AZA combination did not have sufficient studies to explore it.

Reviewer #3:

In this article, Colunga-Lozano et al. perform a thorough and systematic retrospective review that aims to compare the efficacy and safety of low-intensity antileukemic therapy in newly diagnosed acute myeloid leukemia (AML) patients aged 55 years and above that are not candidates for intensive therapy. Although the results obtained in this study do not provide additional insights to the current ASH guidelines, the effort that the authors have made to compile, analyze, and summarize the available data is remarkable and worth to be acknowledged.

However, the authors should consider addressing possible sex-dependent effects in their analysis, which might unveil previously overlooked results in the analyzed data. AML incidence is higher in males than in females, with an age-dependent progressive increase in this difference (Cartwright et al, 2002, doi: 10.1046/j.1365-2141.2002.03750.x). Moreover, male patients show significantly worse outcomes compared to female patients (Hossain et al, 2015, doi: 10.1016/j.canep.2015.10.020) and a sex bias has been also identified in mutational profiles of AML (Juliusson et al, 2020, doi: 10.1182/bloodadvances.2019001335; Metzeler et al, 2016, doi: 10.1182/blood-2016-01-693879; Loghavi et al, 2014, doi: 10.1186/s13045-014-0074-4). Although these evidences illustrate the relevance of gender perspective in the analysis of AML clinical data, sex-specific considerations are currently not made when addressing AML management in the clinic. In this context, the omission of gender-disaggregated data in research studies perpetuate this gender gap in the therapeutic assessment of the patients. Thus, authors might want to provide detailed gender-disaggregated data (at least in the cases of moderate to high certainty evidence), which could potentially identify gender-biased differences in the efficacy and safety of the analyzed therapies and provide additional insights that could have an impact on AML patient management.

Thanks for your suggestion. We agree with your comment, and based on it we revised our body of evidence to identify gender sub-group analyses in the comparisons with moderate certainty of the evidence. After a careful examination we concluded the following. From all the comparisons we included, we classified fifteen comparisons to have moderate certainty of evidence, from which, only two were related to efficacy outcomes (Overall survival; N=2 RCTs, Azacitidine monotherapy vs LDAC monotherapy. Complete remission; N=1 RCT, Azacitidine monotherapy vs Azacitidine combination). From the two studies included in the first comparison, only one of them reported a subgroup analysis based on gender (Dombret 2015). However, the comparisons performed by the authors did not aim the treatment group we included in our review (Azacitidine vs Conventional care regimens). The second comparison did not perform a gender subgroup analysis for the outcome of interest (DiNardo 2020). Regarding the comparisons from the safety outcomes, neither explore a sub-group effect based on gender. We consider an important issue the suggestion raised by the reviewer. However, with our current approach will not be able to properly explore the suggestion, as the comparisons with more studies were classified as low and very low certainty of evidence.
